# Short-term safety and reactogenicity of same-day COVID-19 and influenza vaccination in very old, community-dwelling adults

**DOI:** 10.1007/s41999-026-01477-z

**Published:** 2026-04-18

**Authors:** Robin Šín, Alena Lochmannová, Gizell Green, Miroslav Kubiska

**Affiliations:** 1 Department of Infectious Diseases and Travel Medicine, Faculty of Medicine in Pilsen, Charles University, Alej Svobody 1655/76, Plzeň, 323 00 Czech Republic; 2 University Hospital in Pilsen, Plzeň, Czech Republic; 3 Nursing Department, School of Nursing, Max Stern Yezreel Valley College, Mizra, Israel; 4 Shoham Medical Center, Derech Hanadiv, Pardes Hana Karkur, Israel

**Keywords:** COVID-19 vaccines, Influenza vaccines, Drug-related side effects and adverse reactions, Aged, 80 and over, Immunization schedule

## Abstract

**Purpose:**

To assess short-term safety after routine same-day seasonal influenza and COVID-19 vaccination in community-dwelling adults aged 65 years and older, predominantly very old, and to identify factors associated with adverse events within 48 h.

**Methods:**

We conducted a prospective observational cohort study in a hospital-based infectious diseases department and a collaborating outpatient clinic in the Czech Republic, enrolling adults aged 65 years and older vaccinated between 1 September and 30 November 2025. Participants completed 48 h symptom diaries and measured body temperature at 24 and 48 h. We compared local reactions between vaccine injection sites within individuals and used regression models to examine demographic and clinical correlates of adverse events and the predictive value of 24 h temperature.

**Results:**

The cohort included 169 participants, 75.1% of whom were women, with a mean age of 84.3 years and substantial multimorbidity. Within 48 h, 44.4% reported at least one adverse event, most reactions were mild, and no participant had fever at 48 h. Local reactions were more frequent at the COVID-19 than the influenza injection site. Higher comorbidity count predicted any adverse event (odds ratio 1.3, 95% CI 1.0–1.6), and prior COVID-19 infection predicted systemic adverse events (odds ratio 2.2, 95% CI 1.1–4.4). Higher body temperature at 24 h was associated with higher counts of total, systemic, and local adverse events at 48 h, with the strongest association observed for systemic symptoms (β = 0.4, p < 0.001).

**Conclusion:**

Same-day influenza and COVID-19 vaccination was generally well tolerated in very old, medically complex adults, supporting coadministration with anticipatory counselling for those with higher comorbidity burden or prior COVID-19 infection.

**Supplementary Information:**

The online version contains supplementary material available at 10.1007/s41999-026-01477-z.

## Key summary points

**Aim:**

To examine short-term reactions after same-day influenza and COVID-19 vaccination in older adults living at home.

**Findings:**

Most reactions were mild and short-lived. Local reactions were more common after the COVID-19 vaccine than after the influenza vaccine, and adults with more health problems or a previous COVID-19 infection were more likely to report symptoms.

**Message:**

Same-day influenza and COVID-19 vaccination was generally well tolerated in very old adults living at home and may help support practical vaccine delivery in this group.

**Supplementary Information:**

The online version contains supplementary material available at 10.1007/s41999-026-01477-z.

## Introduction

Older adults bear a disproportionate burden of severe complications and death from both seasonal influenza and COVID-19 [1–4]. Among adults aged 65 years and older, influenza accounts for most related hospitalisations and deaths [2, 3, 5, 6], while vaccination reduces the risk of serious complications and mortality [7, 8]. SARS-CoV-2 infection in this age group is likewise associated with markedly higher risks of hospitalisation, intensive care admission and death [9, 10], although current vaccines substantially attenuate these risks even in very old and multimorbid populations [1, 11, 12]. Following the resumption of respiratory virus circulation after the relaxation of non-pharmaceutical interventions [13], many countries have prioritised older adults for annual influenza vaccination and updated COVID-19 boosters [14, 15].

Influenza and COVID-19 vaccination campaigns in many countries now run in parallel [16, 17], and coadministration during a single visit has been proposed to improve coverage and reduce pressure on health services [16, 18, 19]. Phase 4 trials and observational studies have shown that concomitant administration of COVID-19 and age-appropriate influenza vaccines is immunogenic and does not raise major safety concerns, although local and systemic reactogenicity may be modestly higher than with COVID-19 vaccination alone [20–23]. Systematic reviews likewise indicate that same-day delivery of adult vaccines, including mRNA COVID-19 and seasonal influenza vaccines, is feasible, has an acceptable safety profile, and may streamline service delivery, and many national programmes now allow or actively promote such coadministration [17, 19, 24, 25].

Short-term post-vaccination reactions remain an important concern for both patients and providers. Large COVID-19 vaccine cohorts show that reactogenicity varies by age, sex, vaccine type, and prior SARS-CoV-2 infection [26–29], with younger adults and women generally reporting more local and systemic symptoms than older men [30, 31]. Older adults often report fewer and milder systemic reactions after mRNA vaccination [32, 33], a pattern linked to age-related changes in innate and adaptive immunity described as immunosenescence [34–36]. Because concerns about side effects contribute to vaccine hesitancy in older adults [37, 38], some clinicians and patients prefer to separate influenza and COVID-19 vaccination into two visits despite limited evidence to guide this decision in very old and medically complex adults [18, 39]. In gerontological research, older adults are often stratified into young-old (65–74 years), middle-old (75–84 years), and oldest-old (≥ 85 years) [40, 41]. This reflects the heterogeneity of later life, as the highest age bands differ from younger older adults in frailty, comorbidity, and functional dependence [42]. However, older (middle-old) and especially very old (oldest-old) adults remain underrepresented in several vaccine trial settings [43, 44].

Despite reassuring data from clinical trials [17] and mixed-age observational cohorts [45], important knowledge gaps remain. Very old adults and people with extensive multimorbidity are still under-represented in COVID-19 vaccine studies and most coadministration research, limiting extrapolation to those at highest absolute risk. Evidence on short-term reactogenicity after same-day influenza and COVID-19 vaccination in community-dwelling adults in the highest age bands is scarce, and little is known about how age, sex, comorbidity burden, previous SARS-CoV-2 infection, or allergy history shape local and systemic reactions in this group [17, 24, 46]. Community-dwelling very old adults deserve separate attention because they are a key target group for routine outpatient vaccination and differ from institutionalised older adults in frailty, care arrangements, symptom monitoring, and post-vaccination self-management at home [47]. Early post-vaccination body temperature has rarely been examined as a marker of subsequent reactogenicity in older adults. Clarifying these issues is important both for counselling older patients and for planning vaccination strategies that include very old and medically complex individuals.

In this context, we conducted a prospective observational cohort study of routine same-day seasonal influenza and COVID-19 vaccination in community-dwelling adults aged 65 years and older, with a predominance of participants aged 85 years and older and individuals with multiple chronic conditions.

Our primary objective was to characterise the short-term safety profile of same-day influenza and COVID-19 vaccination by quantifying the frequency and severity of local and systemic adverse events within 48 h in a real-world cohort, rather than to compare coadministration with separate-day vaccination. Secondary objectives were to compare local reactogenicity at the influenza and COVID-19 injection sites, to identify demographic and clinical correlates of adverse events, including comorbidity burden and prior COVID-19 infection, and to explore whether body temperature at 24 h predicts total, systemic and local adverse events at 48 h. These questions were chosen to provide population-specific real-world data for very old adults who remain under-represented in coadministration studies and to clarify how existing coadministration recommendations apply to this clinically vulnerable population.

## Methods

### Study design and setting

This prospective observational study was conducted in adults aged 65 years and older who received routine influenza and COVID-19 vaccination between 1 September and 30 November 2025. Vaccinations and study procedures took place at the Department of Infectious Diseases and Travel Medicine of the University Hospital Pilsen and at a collaborating general outpatient clinic in Planá in the Czech Republic.

### Ethical considerations

The study was conducted in accordance with the Declaration of Helsinki and national regulations on research involving human participants. The protocol, project description and informed consent form were reviewed and approved by the Ethical Committee of the University Hospital Pilsen and the Faculty of Medicine in Pilsen, Charles University, under reference number 130/25.

### Participants and procedure

Eligible participants were community-dwelling older adults aged 65 years and older who were scheduled to receive both influenza and COVID-19 vaccines during the study period. Although eligibility began at age 65 years, no individuals aged exactly 65 years were included in the final sample because participants were recruited consecutively at the vaccination sites during the study period, rather than excluded by design. Participation was voluntary and did not influence access to health care. Participants were excluded if they refused or were unable to provide informed consent, or if they did not receive both vaccines during the same visit.

Before enrolment, all participants received written and oral information about the aims and procedures of the study and about data protection. Those who agreed to participate signed an informed consent form.

Each participant received both vaccines during a single visit. Vaccines were administered intramuscularly into the deltoid muscles by trained medical staff in accordance with current national recommendations and routine practice at the study sites. The two vaccines were injected into opposite arms during the same appointment.

At baseline, immediately before vaccination, participants completed a brief paper questionnaire with assistance from study staff when needed. The questionnaire recorded age, sex, number of chronic comorbidities, history of laboratory-confirmed COVID-19 infection and history of medication allergies. Chronic comorbidities were assessed by self-report using a predefined checklist of 11 conditions as arterial hypertension, chronic ischaemic heart disease, arrhythmia, diabetes mellitus, stroke, etc. For each condition, participants indicated whether they had been diagnosed (yes/no). The total number of endorsed conditions was used as the comorbidity count variable in all analyses.

Body temperature was measured by trained nursing staff at 24 and 48 h after vaccination using a standardised digital thermometer (True Life Care T3). They also completed a symptom diary for 48 h post-vaccination. Local adverse events included pain, swelling or induration and redness at each injection site. Systemic adverse events included allergy, fatigue, weakness, chills, rigors, loss of appetite, headache, myalgia, arthralgia, nausea, vomiting, abdominal pain and diarrhea. For each time point, participants indicated whether each symptom was present. Because the diary captured symptom presence rather than intensity, adverse event severity was inferred indirectly from the number of concurrently reported symptoms, with higher counts interpreted as greater symptom burden.

From the symptom diary we constructed three count variables: the total number of adverse events, the number of systemic adverse events and the number of local adverse events recorded within 48 h after vaccination, with higher values indicating more concurrent symptoms. We also created a binary variable for the presence of any adverse event, coded as 1 if the participant reported at least one local or systemic symptom and 0 if no symptoms were reported. This binary indicator was used as the primary outcome. The three count variables were used as secondary outcomes. For descriptive purposes, overall adverse event burden was classified into four categories based solely on the total number of reported symptoms: none (0 symptoms), mild (1–2 symptoms), moderate (3–4 symptoms) and severe (≥ 5 symptoms). These categories reflect the number of concurrent symptoms and do not represent a clinical severity grading based on symptom intensity, duration or functional impact. Body temperature at 24 and 48 h post-vaccination was analysed as a supplementary objective marker, while systemic symptoms were captured separately in the participant diary.

### Statistical analysis

Data were entered into a secure database and analysed using IBM SPSS Statistics. Descriptive statistics were used to summarise baseline characteristics and the frequency and distribution of adverse events. For descriptive and stratified analyses, age was categorised into three predefined groups: young-old (65–74 years), middle-old (75–84 years) and oldest-old (≥ 85 years), following the widely used geriatric classification that reflects clinically meaningful differences in functional capacity, frailty and healthcare needs across age bands in later life [48]. For regression analyses, age was entered as a continuous variable to maximise statistical power. Categorical variables are presented as counts and percentages, and continuous variables as means with standard deviations or medians with interquartile ranges, as appropriate. Chi-square tests were used to compare categorical variables between groups.

The McNemar test was used for paired comparisons, as each participant received both vaccines simultaneously in different arms, which allowed within-subject correlation to be considered when comparing vaccine-specific local reactions.

Binary logistic regression was used to examine the association between each predictor variable and the occurrence of any adverse event within 48 h. A dichotomous outcome variable was created, coded as 1 for participants experiencing at least one symptom and 0 for those with no symptoms. Each potential risk factor—age, sex, comorbidity count, history of COVID-19 infection and history of medication allergies—was entered separately into individual logistic regression models using the ENTER method to assess crude associations. Variables reaching a significance level of p < 0.05 in univariate analysis were subsequently considered for multivariable analysis using forward stepwise selection with an entry criterion of p < 0.05. All five candidate predictors were entered simultaneously into each model, allowing potential confounding by age and sex to be evaluated.

Three separate linear regression analyses were conducted to examine the predictive value of body temperature at 24 h post-vaccination on adverse events at 48 h. The total number of adverse events, the number of systemic adverse events and the number of local adverse events at 48 h were used as continuous outcome variables, with body temperature at 24 h entered as the predictor using the ENTER method.

All tests were two-sided and p values below 0.05 were considered statistically significant.

## Results

The study included 169 participants with a mean age of 84.3 years (SD = 7.6, range: 66–98 years). Although eligibility started at age 65 years, no individual aged exactly 65 years was recruited during the study period. Table 1 presents additional demographic and clinical characteristics of the study population.

**Table 1:** Demographic and clinical characteristics of study participants and adverse event distribution following concurrent COVID-19 and influenza vaccination

Characteristic	n (%)
Demographics	
Age, mean ± SD	84.3 ± 7.6
*Sex*	
Female	127 (75.1)
*Comorbidities*	
No comorbidities	16 (9.5)
One comorbidity	49 (29.0)
Two comorbidities	30 (17.8)
Three or more comorbidities	74 (43.8)
*Prior infection history*	
No prior infection	123 (72.8)
Prior COVID-19 only	46 (27.2)
Prior influenza only	0 (0.0)
*Adverse events systemic and local*	
Systemic AE severity distribution	
None	117(69.2)
Mild (1–2 symptoms)	38 (22.5)
Moderate-severe (≥ 3 symptoms)	14 (8.3)
*Local injection site reactions*	
Influenza vaccine pain	4 (2.4)
Influenza vaccine Swelling	9 (5.3)
*Local COVID-19 vaccine injection site reactions*	
COVID-19 vaccine Pain	31 (18.3)
COVID-19 vaccine Swelling	24 (14.2)
*Overall adverse event severity*	
None (no AE)	94 (55.6)
Mild (1–2 total symptoms)	52 (30.8)
Moderate-severe (≥ 3 total symptoms)	23 (13.6)

Participants could experience multiple concurrent adverse events

*AE* Adverse event, *COVID-19* Coronavirus disease 2019

Table 1 shows that the cohort was predominantly female (75.1%) and had a high comorbidity burden, with 43.8% having three or more chronic conditions and only 9.5% having no comorbidities. Prior COVID-19 infection was documented in 27.2% of participants, while no participants reported prior influenza infection. Regarding systemic adverse events, 69.2% experienced none, 22.5% had mild symptoms (1–2 symptoms), and 8.3% had moderate-severe reactions (≥ 3 symptoms). Local injection site reactions were more common for COVID-19 vaccine compared to influenza vaccine, with COVID-19 vaccine causing pain in 18.3% and swelling in 14.2% of participants, while influenza vaccine caused pain in only 2.4% and swelling in 5.3%. When classified by overall severity, 55.6% experienced no adverse events, 30.8% had mild reactions (1–2 total symptoms), and 13.6% had moderate-severe reactions (≥ 3 symptoms).

### Safety profile and incidence of adverse events following same-day COVID-19 and influenza vaccination in adults aged 65 and older

Table S1 shows that, overall, 44.4% of participants experienced at least one adverse event, with systemic reactions occurring in 30.8% and local injection site reactions in 23.7%. The mean number of total adverse events per participant was 0.9 (± 1.3), comprising an average of 0.5 (± 0.9) systemic symptoms and 0.4 (± 0.8) local reactions.

Table 2 shows that systemic reactions were predominantly mild, with headache (14.8%) and fatigue (11.8%) being most common. Notably, no participant developed fever at 48 h post-vaccination. Local reactions showed vaccine-specific differences, with COVID-19 injection sites demonstrating higher rates of both pain (18.3%) and swelling (14.2%) compared to influenza sites (2.4% and 5.3%, respectively).

**Table 2:** Specific adverse events frequency (N = 169)

Adverse event	n (%)
*Systemic reactions*	
Headache	25 (14.8)
Fatigue	20 (11.8)
Chills	13 (7.7)
Myalgia	11 (6.5)
Nausea	8 (4.7)
Vomiting	8 (4.7)
Weakness	4 (2.4)
Fever (≥ 37.5°C) at 48h	0 (0.0)
*Local injection site reactions*	
COVID-19 injection site pain	31 (18.3)
COVID-19 injection site swelling	24 (14.2)
Influenza injection site swelling	9 (5.3)
Influenza injection site pain	4 (2.4)

Adverse event severity, defined by symptom count as described in the Methods section, is presented in Table 3.

**Table 3:** Severity adverse events distribution by demographics

Characteristic	None n (%)	Mild n (%)	Moderate n (%)	Severe n (%)	p-value
Overall	94 (55.6)	52 (30.8)	21 (12.4)	2 (1.2)	
*Sex*					0.03
Male (n = 42)	19 (45.2)	17 (40.5)	4 (9.5)	2 (4.8)	
Female (n = 127)	75 (59.1)	35 (27.6)	17 (13.4)	0 (0.0)	
*Age group*					< 0.001
65–74 years (n = 13)	9 (69.2)	2 (15.4)	0 (0.0)	2 (15.4)	
75–84 years (n = 61)	25 (41.0)	29 (47.5)	7 (11.5)	0 (0.0)	
85 + years (n = 95)	60 (63.2)	21 (22.1)	14 (14.7)	0 (0.0)	

Severity categories: None = no adverse events; Mild = 1–2 symptoms; Moderate = 3–4 symptoms; Severe =  ≥ 5 symptoms. Chi-square test is used for comparisons. p < 0.05 indicates statistically significant differences in severity distribution between groups

Table 3 shows that most participants (55.6%) experienced no adverse events, with progressively fewer experiencing mild (30.8%), moderate (12.4%), or severe reactions (1.2%). Significant differences were observed by sex (p = 0.026) and age (p < 0.001), with the 75–84 age group showing the highest proportion of mild reactions.

Table 4 shows that COVID-19 vaccination sites demonstrated significantly higher local reactogenicity compared to influenza sites McNemar test p < 0.001 for both pain and swelling, with an approximately fourfold higher rate of any local reaction.

**Table 4:** Comparison of local reactions by vaccine type

Local reaction	COVID-19 vaccine	Influenza vaccine	p-value
Injection site pain	31 (18.3%)	4 (2.4%)	< 0.001
Injection site swelling	24 (14.2%)	9 (5.3%)	< 0.001
Any local reaction	37 (21.9%)	9 (5.3%)	< 0.001

*McNemar test for paired comparisons, p < 0.05 indicates statistically significant differences

### Clinical (comorbidity, prior COVID-19 and medication allergies) and demographic factors (age and sex) predicting adverse events following concurrent vaccination COVID-19 and influenza vaccination in adults aged 65 and older

To identify predictors of adverse events (defined as the presence of one or more symptoms yes/no), univariate binary logistic regression analyses were performed for each potential risk factor (see Table 6).

Table 5 shows that comorbidity count (OR = 1.3, p = 0.04) was significantly associated with any adverse event. Prior COVID-19 infection showed non-significance trend toward higher odds of any adverse event (OR = 2.0, p = 0.1). Age, sex, and medication allergies were not significant predictors.

**Table 5:** Univariate binary logistic regression analysis for each risk factor for any adverse event (N = 169)

Risk factor	OR	p-value
Age	1.0	0.4
Female sex	0.6	0.1
Comorbidity count (0–11)	1.3	0.04*
Prior COVID-19	2.0	0.1
Medication allergies	1.3	0.6

p < 0.05 indicates statistically significant differences, no participants with prior influenza infection; analysis not possible

In Table S2, three separate forward stepwise selection regression models were conducted for different adverse event outcomes. The same candidate predictors were entered into each model: age, sex, total number of comorbidities, prior COVID-19 infection, and history of medication allergy.

Table S2 shows different predictors emerged as significant for each adverse event outcome. Total number of comorbidities was the sole predictor selected for any adverse event (OR = 1.3, p = 0.04), prior COVID-19 infection uniquely predicted systemic adverse events (OR = 2.2, p = 0.03), while no variables met the significance threshold for local adverse events.

### Early body temperature as a predictor of subsequent adverse events following concurrent COVID-19 and influenza vaccination in adults aged 65 and older

Table 6 shows that body temperature at 24 h post-vaccination demonstrated significant positive associations with all adverse event outcomes at 48 h. The strongest relationship was observed with systemic adverse events (β = 0.4, R^2^ = 0.2, p < 0.001). Total adverse events showed a similarly robust association (β = 0.4, R^2^ = 0.2, p < 0.001). The relationship with local adverse events, while statistically significant, was considerably weaker (β = 0.2, R^2^ = 0.03, p = 0.04), suggesting that early fever response is a stronger predictor of systemic rather than local reactions.

**Table 6:** Association between body temperature at 24 hours and adverse events at 48 hours post-vaccination (N = 169)

Outcome variable	B	SE	β	R^2^	p-value
Total adverse events (0–12)	1.5	0.3	0.4	0.2	< 0.001
Systemic adverse events (0–8)	1.1	0.2	0.4	0.2	< 0.001
Local adverse events (0–4)	0.4	0.2	0.2	0.03	0.04

*B* Unstandardized coefficient, *SE* Standard error, *β* Standardized coefficient, *CI* confidence interval, *R*^*2*^ Coefficient of determination

## Discussion

In this real-world cohort of very old adults with a high burden of chronic disease, same-day administration of seasonal influenza and COVID-19 vaccines was generally well tolerated. Roughly half of participants reported any adverse event within 48 h, most events were mild and captured within the 48-h follow-up window, and no participant developed fever at 48 h. Local reactions were more frequent at the COVID-19 than at the influenza injection site but remained clinically manageable. These findings add descriptive real-world evidence on the short-term tolerability of coadministration [20, 49] in a population that is often under-represented in vaccine trials [44] yet at highest risk of severe outcomes from both influenza and COVID-19 [50, 51].

Our results are consistent with clinical trials and observational studies that evaluated concomitant COVID-19 and influenza vaccination in younger or more heterogeneous adult populations. Phase 4 studies, including the ComFluCOV trial, showed that concomitant administration of COVID-19 vaccines with age-appropriate influenza vaccines did not raise new safety concerns, although local and systemic reactogenicity was slightly increased compared with COVID-19 vaccination alone [20, 23, 24]. Systematic reviews have concluded that coadministration of adult vaccines, including mRNA COVID-19 vaccines and seasonal influenza vaccines, is both safe and operationally advantageous [17, 19, 39]. Compared with these reports, our cohort was substantially older and had a high comorbidity burden, yet the overall frequency and pattern of adverse events remained within the previously reported range. These findings suggest that trial data may also be applicable to very old, multimorbid adults in everyday practice, although confirmation in larger controlled studies is still needed.

We observed clear vaccine-specific differences in local reactogenicity, with COVID-19 injection sites showing higher rates of pain and swelling than influenza sites. This pattern mirrors earlier work in which COVID-19 mRNA vaccines were consistently more reactogenic at the injection site than inactivated influenza vaccines, both when administered alone and when coadministered [22, 52, 53]. In our study, these local reactions were confined to the 48-h observation window and were not predicted by demographic or clinical characteristics, which supports the view that they primarily reflect intrinsic properties of the vaccine formulations and their adjuvants rather than modifiable patient factors.

The distribution of overall adverse event severity differed by age and sex, although subgroup counts were small. In broader COVID-19 vaccine cohorts, younger age and female sex have generally been associated with higher reactogenicity. In our very old population, the oldest age group did not exhibit the greatest symptom burden. The highest proportion of mild reactions was observed in those aged 75–84 years, while many adults aged ≥ 85 years reported no symptoms. This pattern is compatible with literature on immunosenescence, where older adults often report fewer and milder systemic reactions after mRNA vaccination, [32, 54] which may reflect a dampened inflammatory response.

Comorbidity burden and prior COVID-19 infection emerged as relevant correlates of reactogenicity. A higher number of chronic conditions was associated with an increased likelihood of any adverse event, and previous SARS-CoV-2 infection predicted systemic reactions in multivariable models. Similar patterns have been reported in studies of COVID-19 vaccination alone, where pre-existing morbidity [55, 56] and a history of infection [57, 58] were linked to more frequent or more intense self-reported symptoms. The mechanisms are likely multifactorial, involving altered baseline inflammation, heightened symptom awareness and differences in immune priming. For clinicians, these patterns underline the importance of anticipatory counselling in older adults with multiple comorbidities and in those who have recovered from COVID-19, while also emphasising that even in these subgroups most reactions remained mild.

A distinctive feature of this study is the use of early post-vaccination body temperature as a simple physiological marker. Higher temperature at 24 h was associated with more systemic and total adverse events at 48 h, whereas the association with local reactions was weaker. This is consistent with work suggesting that systemic reactogenicity, particularly fever or subfebrile responses, can reflect a stronger inflammatory response to mRNA vaccination [59–62]. Some studies have reported modest positive correlations between systemic symptoms and antibody titres [62, 63], while others have not found a clear relationship [64, 65], and the clinical relevance of these associations remains uncertain. We did not measure antibody responses, so we cannot infer anything about protection. However, our findings suggest that simple home temperature monitoring may help to identify those older adults who are more likely to experience a more pronounced cluster of systemic symptoms after concurrent vaccination, which may be useful for planning short-term support and follow-up contacts in frailer patients.

From a public health perspective, these findings are consistent with current recommendations that allow or encourage same-day administration of influenza and COVID-19 vaccines in older adults and highlight the potential programme advantages of delivering both vaccines together [16, 17, 39]. Our study adds population-specific real-world evidence from very old, community-dwelling adults with substantial comorbidity, a group that remains under-represented in prior coadministration studies and shows that concurrent vaccination was feasible and generally well tolerated in this high-priority cohort. This may help to reassure patients and providers who are hesitant about receiving or offering both vaccines on the same day.

This study provides real-world evidence that same-day administration of influenza and COVID-19 vaccines is generally well tolerated in very old adults with multiple comorbidities. However, several limitations should be considered when interpreting these findings. The sample size was modest, and the participants were recruited from a limited number of clinical settings within a single region, which restricts generalisability, reduces statistical power, and means that rare but clinically significant adverse events may not have been captured. The observational design and the absence of a comparison group receiving the vaccines on separate days also prevent direct conclusions about the relative reactogenicity of coadministration versus separate administration. Adverse events were recorded by participants using symptom diaries, so reporting bias cannot be excluded, and the 48 h follow-up window did not capture delayed reactions or later-evolving symptoms. Although the diary captured a broad range of subjective systemic symptoms, including fatigue, weakness, headache, myalgia, arthralgia, and gastrointestinal complaints, it did not assess cognitive changes or other geriatric manifestations that may also be clinically relevant. Because the diary did not capture symptom intensity, severity was approximated from the number of reported symptoms rather than assessed directly. We focused on short-term reactogenicity and did not assess immunogenicity or clinical effectiveness, which prevents any conclusions about the relationship between symptoms, antibody responses and protection. In addition, the multivariable models were exploratory and may be affected by residual confounding and overfitting. Taken together, these constraints mean that our findings should be interpreted as hypothesis-generating and as complementary to, rather than a substitute for, larger controlled studies.

## Conclusions

In this prospective cohort of very old, community-dwelling adults with a high burden of chronic disease, routine same-day administration of seasonal influenza and COVID-19 vaccines was generally well tolerated. Although almost half of participants reported at least one local or systemic adverse event within 48 h, reactions were mostly mild and no fever was observed at 48 h. Local reactions were more frequent at the COVID-19 than at the influenza injection site but remained clinically manageable. Comorbidity burden and prior COVID-19 infection were associated with a higher likelihood of reporting adverse events, and early post-vaccination body temperature was positively related to the intensity of subsequent local and systemic symptoms.

These findings provide population-specific real-world descriptive evidence suggesting that same-day influenza and COVID-19 vaccination is generally well tolerated in very old and medically complex adults, although larger comparative studies are needed to assess uncommon adverse events, compare tolerability with separate-day administration, and determine how far these findings can be generalised. They are consistent with existing recommendations that allow or encourage coadministration in older age groups and offer clinicians concrete information for counselling patients about expected short-term reactions. At the same time, the single-country setting, modest sample size and absence of a comparison group vaccinated on separate days mean that the results should be interpreted as complementary to larger controlled studies. Further work is needed to confirm these patterns in more diverse populations and to examine how short-term reactogenicity relates to immunogenicity and clinical protection in very old adults.

## Supplementary Information

Below is the link to the electronic supplementary material.

Supplementary file1 (DOCX 15 KB)

Supplementary file2 (DOCX 15 KB)

## Data Availability

Data available on reasonable request from the corresponding author.
