# Supplementary material for: Short-term safety and reactogenicity of same-day COVID-19 and influenza vaccination in very old, community-dwelling adults
Source: Eur Geriatr Med. 2026 Apr 18;17(3):1523–33. doi: 10.1007/s41999-026-01477-z (PMC13309411; doi:10.1007/s41999-026-01477-z)
Supplement: Supplementary file 1 — Supplementary file1 (DOCX 15 KB) [file 41999_2026_1477_MOESM1_ESM.docx]

**Sup S1:** Overall Incidence of Adverse Events (N=169)

| **Adverse Event Category** | **n (%)** | **Mean ± SD** |
| --- | --- | --- |
| Any adverse event | 75 (44.4) | - |
| Any systemic adverse event | 52 (30.8) | - |
| Any local adverse event | 40 (23.7) | - |
| Total number of adverse events | - | 0.9 ± 1.3 |
| Systemic adverse events score | - | 0.5 ± 0.9 |
| Local reactions score | - | 0.4 ± 0.8 |

*Note: SD, standard deviation; Mean ± SD represents the average number of events per participant with the standard deviation. Participants could experience multiple concurrent adverse events across different categories.*
