# Supplementary material for: Short-term safety and reactogenicity of same-day COVID-19 and influenza vaccination in very old, community-dwelling adults
Source: Eur Geriatr Med. 2026 Apr 18;17(3):1523–33. doi: 10.1007/s41999-026-01477-z (PMC13309411; doi:10.1007/s41999-026-01477-z)
Supplement: Supplementary file 2 — Supplementary file2 (DOCX 15 KB) [file 41999_2026_1477_MOESM2_ESM.docx]

**Sup S2:** Stepwise Forward Selection Models for Adverse Events

| **Outcome/Selected Predictors** | **OR** | **95% CI** | **p-value** |
| --- | --- | --- | --- |
| Model 1: Any Adverse Event 48 |  |  |  |
| Total Number of Comorbidities (0-11) | 1.3 | 1-1.6 | 0.04* |
| Variables not selected: Prior COVID-19 (p=0.1), Sex (p=0.1), Age (p=0.2) |  |  |  |
| Model 2: Systemic Adverse Events 48 |  |  |  |
| Prior COVID-19 | 2.2 | 1.1-4.4 | 0.03* |
| Variables not selected: Comorbidities (p=0.1), Age (p=0.6), Sex (p=0.8) |  |  |  |
| Model 3: Local Adverse Events 48 |  |  |  |
| No predictors met entry criteria | - | - | - |
| Variables not selected: Sex (p=0.1), Age (p=0.2), Comorbidities (p=0.5), Prior COVID-19 (p=0.7) |  |  |  |

Note: *p<0.05; Medication allergy history was not significant in any model (all p>0.25)
